# Parent-Reported Oral Health Status and Dental Care Habits in Primary Schoolchildren: A Cross-Sectional Study

**DOI:** 10.3390/children13101342

**Published:** 2026-10-02

**Authors:** Loredana-Cristina Dumitrașcu, Raluca-Paula Vacaru, Ruxandra Ionela Sfeatcu, Ilie-Andrei Condurache, Arina Vinereanu

**Affiliations:** 1 Oral Health and Community Dentistry Department, Faculty of Dentistry, Carol Davila University of Medicine and Pharmacy, 041292 Bucharest, Romania; loredana.dumitrascu@umfcd.ro (L.-C.D.); ruxandra.sfeatcu@umfcd.ro (R.I.S.); 2 Embryology and Microbiology Department, Faculty of Dentistry, Carol Davila University of Medicine and Pharmacy, 041292 Bucharest, Romania; 3 Dr. Victor Babeș Clinical Hospital of Infectious and Tropical Diseases, 030303 Bucharest, Romania; ilie.condurache@spitalulbabes.ro; 4 Pedodontics Department, Faculty of Dentistry, Carol Davila University of Medicine and Pharmacy, 041292 Bucharest, Romania; arina.vinereanu@umfcd.ro

**Keywords:** pediatric oral health, dental caries prevention, parent education, health behavior

## Abstract

**Highlights:**

**What are the main findings?**

- Although parents generally perceived their children’s oral health favorably, dental service utilization remained predominantly low or in response to an existing problem, with non-attendance and pain-driven dental service utilization linked to rural living and lower parental education.
- Unfavorable parent-reported oral health habits, including lack of fluoridated toothpaste, poor brushing frequency, and daily cariogenic dietary intake were significantly associated with increased dental pain and symptomatic dental care-seeking.

**What are the implications of the main findings?**

- Public health interventions should focus toward sustainable preventive approaches by providing targeted, actionable guidance on routine dental visits, pain-free dental treatments, daily hygiene, and sugar reduction.
- Educational outreach and policy efforts should specifically target rural communities and lower-education households to bridge health literacy gaps and promote equitable access to preventive pediatric dental care.

**Abstract:**

**Background/Objectives**: Pediatric oral health during primary school years is linked to systemic and psychosocial development, influenced by parental health habits. The aim of this study was to evaluate parent-reported oral health status, oral hygiene habits, and dietary patterns in primary schoolchildren, as well as parental awareness regarding the impact of oral health on children’s overall wellbeing. **Methods**: A cross-sectional study was conducted, administering an online questionnaire to 1870 parents of primary schoolchildren, which evaluated the parent’s knowledge, behaviors and attitudes regarding the oral health and diet of their children. **Results**: Parent-reported child oral and gum health was overall favorable, with daughters (*p* < 0.01 for gum health) and children of parents with higher educational background (*p* < 0.001) receiving higher ratings. While toothbrushing with fluoridated toothpaste was widely reported, fluoride usage and dental flossing remained heavily stratified socio-demographically (*p* < 0.001). The consumption of fruits and cariogenic snacks was frequent and soft drink intake demonstrated an age-dependent increase (*p* = 0.001). In total, 42.4% of parents reported that their children rarely experienced dental pain; increased pain frequency was associated with poorer parent-reported oral health ratings and cariogenic diets (*p* < 0.001). Child dental service utilization was predominantly low and remained symptom- or treatment-driven, with non-attendance and pain-triggered dental visits significantly linked to rural residence, lower parental academic attainment, poor brushing frequency and cariogenic diets (*p* < 0.001). **Conclusions**: Despite favorable parent-reported oral health of primary schoolchildren, dental pain, alongside functional and psychosocial impacts of oral diseases were reported, and dental visits were predominantly symptom- or treatment-driven.

## 1. Introduction

Optimal maintenance of oral health in primary schoolchildren aged 6 to 12 years old constitutes a fundamental determinant of their future physiological development and psychosocial wellbeing [1]. The mixed dentition phase is of particular clinical significance, as it is characterized by the simultaneous presence of deciduous teeth, with their associated dental pathologies, and the eruption of permanent teeth, which are particularly vulnerable to carious lesions in the first years post-eruption [2,3]. Poor oral hygiene during this developmental phase extends beyond the associated symptomatology of localized odontogenic pathologies [4]. Systemic consequences may include sleep deprivation and nutritional compromises that may have an effect on general development [5,6]. Beyond these physiological concerns, the consequences may be related to both aesthetic and functional aspects of dentition, affecting phonation and psychosocial behavior, eventually manifesting as lowered self-perception, increased school absenteeism and poor quality of life [7,8].

Parental involvement is the primary driver in establishing healthy oral hygiene and dietary habits during childhood [9]. By shaping the home environment, parents act as both facilitators and role models, and their active supervision directly influences how consistently and effectively a child removes plaque and maintains oral health [10]. Furthermore, parents play an essential role in managing cariogenic risk by controlling sugar intake and establishing structured eating routines. For a child to adopt lifelong oral health routines, parents must successfully translate their own dental knowledge into daily practice, effectively reducing the likelihood of early dental disease [11].

The periodicity and motivation surrounding children’s dental visits are highly mediated by parental influence, wherein transmitted dental anxiety and reactive, pain-driven care-seeking directly compromise preventive, routine access of dental services. In alignment with this need, the Erasmus+ PaFein+ project aims to create positive oral health attitudes and prevent dental anxiety in pediatric populations [12]. As part of this initiative, a dedicated Parents’ Section was established to provide evidence-based guidance for managing stress-free dental visits, with the present study reflecting key research undertaken within this framework.

The main objective of this study was to evaluate parent-reported oral health status, oral hygiene habits, and dietary patterns in primary schoolchildren. Additionally, the study aimed to assess parental awareness regarding the impact of oral health on children’s overall wellbeing.

## 2. Materials and Methods

### 2.1. Study Design and Participants

This was a cross-sectional study, following STROBE and CHERRIES checklists, part of a broader longitudinal study within the oral health education program “Little Dentists”, 2025 Edition, which was conducted in primary schools in Bucharest and in 20 counties across Romania. This study was approved by the Research Ethics Committee of the “Carol Davila” University of Medicine and Pharmacy Bucharest (no. 13896/5 June 2025). The inclusion criteria are related to the voluntary enrolment of the primary school teachers in the “Little Dentists” program, who further distributed an online questionnaire to the parents of the schoolchildren attending their classes. The exclusion criteria are related to the refusal of participation. A total of 1889 parents of the schoolchildren enrolled in the classes of the 778 primary school teachers that previously enrolled in the “Little Dentists” program accepted the invitation received through contact mode to participate in the study and were sent the online questionnaire. Due to the open-distribution method via schoolteachers, the precise sampling frame, specifically the total number of parents who received the survey invitation, remains unknown. Survey metrics were therefore limited to recording the number of participants who initiated the online questionnaire. Before enrolment, the parents were informed about the aims of the study and gave their consent for participation in the study. The parents who refused to consent for participation in the study were further excluded (*n* = 19), while those who gave their consent and further filled the questionnaire were anonymously included in the study sample (*n* = 1870). Parents were not participants in the educational program and did not receive any oral health educational materials before completing the survey. Although teachers were enrolled in the program, the parent questionnaire was administered independently of the educational intervention, and parents had no direct exposure to program materials before responding. As part of the participation in the program, after filling the online questionnaire, the parents were invited to join an online community initiated and administered by the “Little Dentists” partners, where they received access to useful information about their children’s oral health. Data were collected in compliance with the General Data Protection Regulation (GDPR). Access to the data was restricted to the authors, who were solely responsible for data analysis, and all data were stored securely throughout the study.

### 2.2. The Oral Health Education Program “Little Dentists”

The “Little Dentists” program (“Micii Stomatologi”, as originally entitled in Romanian) is an oral health educational program conducted by the Romanian Red Cross in partnership with the Romanian National Association of Pediatric Dentistry and the Discipline of Oral Health and Community Dentistry within the Faculty of Dentistry at “Carol Davila” University of Medicine and Pharmacy in Bucharest. The “Little Dentists” program aims to provide oral health education to schoolchildren aged 6 to 12 years old, who are enrolled in the first level educational classes, through their primary school educators and their caregivers. Within the program, the three strategic and educational partners developed scientific, evidence-based materials regarding oral health and preventive dental approaches which were provided to primary school teachers, who were further encouraged to use and disseminate the information to schoolchildren attending their classes and to their subsequent families. The teaching and educational materials developed within the project are based on materials provided by the Erasmus+ 2023-1-TR01-KA220-HED-000155608 PaFein+ project team and followed the WHO recommendations [13].

The “Little Dentists” program is designed to be carried out in multiple phases. The objective of the first phase is to evaluate the knowledge, attitudes and behaviors of parents and primary school teachers regarding oral health, routine oral hygiene practices, protective dietary habits, and scheduled prophylactic dental visits of schoolchildren. Problem-driven dental visits, symptom-triggered care-seeking behaviors, and parental responses to existing oral health conditions are further evaluated, in order to measure how parents perceive the impact of both home-routine preventive practices and dental treatments on their children’s overall wellbeing. The second phase of the program aims to provide educational materials and online training to primary school teachers, who will further disseminate the information related to oral health to schoolchildren attending their classes. The online training for teachers is performed by trained dental professionals.

### 2.3. Questionnaire Contents and Distribution

The questionnaire used in the present study was the Romanian version developed and validated by Perpelea et al. (2024), adapted from the World Health Organization Children’s Questionnaire from 2013 [14]. The adaptation and validation process included forward translation, expert review, back-translation, and pilot testing in adults, following World Health Organization methodology [14]. The questionnaire was administered to parents online using Google Forms (Google LLC, Mountain View, CA, USA) and it comprised 17 items adapted for parents, which were mandatory. It evaluated the parent’s knowledge, behaviors and attitudes regarding the oral health and diet of their children. Each completed questionnaire referred to one child enrolled in the participating primary school class. Parents were instructed to complete the questionnaire for one child only, and no parent-level identifier was collected that would allow multiple submissions by the same parent to be linked. If multiple children from the same family could have been represented, this could not be identified from the available dataset. The general data collected through the questionnaire included information about the socio-demographic characteristics of the schoolchildren (Q1–Q4) and the educational levels of the parents (Q16–Q17). The items related to oral health collected data about the parents’ perceptions about their children’s oral health status (Q5), dental pain (Q6), dental visits (Q7–Q8), dental hygiene habits (Q9–Q12), parent-reported oral health impacts (Q13), eating (Q14) and smoking habits (Q15). Items Q5, Q6, Q7, Q8, Q12, Q13, Q16, and Q17 included an answer related to parents’ uncertainty regarding the questioned matter: “I do not know/I cannot estimate” or “I do not know/I cannot remember” or “I do not know”. The questionnaire was distributed online to parents by the primary school teachers enrolled in the program in June 2025, and the responses referred to the children attending the classes of the teacher that distributed the questionnaire to the parents. The complete Romanian questionnaire and an English version are presented in Table S1.

### 2.4. Statistical Analysis

Statistical analyses were performed using IBM SPSS Statistics for Windows, Version 25.0 (IBM Corp., Armonk, NY, USA) and were illustrated using Microsoft Excel 2024 (Microsoft Corporation, Redmond, WA, USA). The “I do not know/I cannot estimate” or “I do not know/I cannot remember” responses were treated as non-informative and excluded item-wise, and analyses involving two variables included only participants with valid responses to both variables. No formal a priori sample-size calculation was performed for this baseline survey. Because the study was conducted within an existing educational program, the analytic sample consisted of all eligible and consenting respondents who completed the questionnaire during the recruitment period. Quantitative variables were tested for normal distribution using the Shapiro–Wilk Test and data distributions were presented as means, standard deviations (SD), medians, quartiles and interquartile ranges. Qualitative variables were expressed as counts or percentages and were tested between groups using Fisher’s Exact Test. Z-tests with Bonferroni correction were used to further detail the results obtained in the contingency tables. Quantitative independent variables with non-parametric distribution were tested between groups using Mann–Whitney U or Kruskal–Wallis H tests (with post hoc Dunn–Bonferroni tests).

Adjustment of *p*-values for multiple testing of primary outcomes (current dental health—Q5, current gum health—Q5, dental pain frequency—Q6, dental visit frequency—Q7, reason for last dental visit—Q8) was determined using the Holm–Bonferroni method while the adjustment for multiple testing of secondary outcomes (Q9–Q15) was determined using the Benjamini–Hochberg method in R software version 4.4.0 (The R Project for Statistical Computing). Besides the association between dental health status and gender which was determined to be unsignificant after the adjustment, all other tested associations remained significant after adjustment.

In order to account for within-cluster correlation, generalized estimating equations were implemented to predict the analyzed outcomes, using an independent structure and a robust estimation of the covariance matrix, with a binomial logistic/ordinal logistic structure, according to the outcome. In cases of the reason for dental visit, due to the violation of proportional-odds assumption, a generalized estimating equation with a multinomial structure was used using the geepack package in R software. Effect estimates for each predictor were calculated as odds ratios with 95% confidence intervals.

The threshold for the significance level was considered to be α = 0.05.

## 3. Results

### 3.1. Socio-Demographic Distribution of Participants

Participant distribution across the sample was relatively balanced: 51.4% of the parents responded for their sons, and 52.4% of the parents were living in an urban area. The mean age of children was 8.48 ± 1.49 years old (95% C.I. = 8.42–8.55, median = 8; IQR = 7–10) and the two largest groups of participants were studying in the third (22%) or second grade (21%). Parents’ education analysis showed that 29.7% of the fathers had academic studies and 50% had high school education, while 41.1% of the mothers had academic studies and 43% had high school education.

### 3.2. Parents’ Perception of Their Children’s Oral Health Status

Parent-reported perceptions of children’s dental and gum health are detailed in Table 1. Excellent ratings of gum health status were more frequently attributed to daughters (*p* < 0.001, Fisher’s Exact Test), while average and good ratings, respectively, were more frequently attributed to sons. Parents’ perception of children’s dental health differed significantly according to the parent’s educational level. Children whose fathers had academic studies were more frequently perceived as having very good ratings of dental health status compared to poor/average/good ratings (40.4% vs. 17%/25.9%/26.7%, *p* < 0.001, Fisher’s Exact Test, Cramer’s V = 0.154, N = 1653). Similarly, children whose mothers had academic studies were more frequently perceived as having very good ratings of dental health status compared with poor/good ratings (52.3% vs. 22.3%/39.5%, *p* < 0.001—Fisher’s Exact Test, Cramer’s V = 0.146, N = 1744). Excellent ratings of children’s dental health status showed no statistical differences regarding the parent’s educational levels. Children whose fathers had academic studies were more associated with very good/excellent ratings of gum health status compared to poor/average/good ratings (38.6%/40.8% vs. 11.1%/18.9%/21.6%; *p* < 0.001, Fisher’s Exact Test, Cramer’s V = 0.203, N = 1663). Children whose mothers had academic studies were more associated with very good/excellent ratings of gum health status compared to poor/average/good ratings (48.5%/48.2% vs. 20.7%/32.4%/36.3%; *p* < 0.001, Fisher’s Exact Test, Cramer’s V = 0.139, N = 1756).

Prediction of dental health status in a GEE model, using predictors which could impact dental health aspects (child age, gender, environment, academic studies for the fathers/mothers, frequency of dental hygiene, usage of fluoride toothpaste and frequency of biscuits, cakes, cream cakes, sweet pies, etc., or soft drinks) reveals that children with fathers that have academic studies have higher odds of a higher rating for the dental health status by 1.417 times (95% C.I. = 1.082–1.855, *p* = 0.011); having a higher dental hygiene frequency increases the odds of a higher rating for all hygiene categories and consuming biscuits, cakes, cream cakes, sweet pies, etc., just once a week increased the odds of a higher rating for the dental health status by 1.569 times (95% C.I. = 1.268–1.942).

Prediction of gum health status in a GEE model, using predictors which could impact dental health aspects (child age, gender, environment, academic studies for the fathers/mothers, frequency of dental hygiene, usage of fluoride toothpaste and frequency of biscuits, cakes, cream cakes, sweet pies, etc., or soft drinks) reveals that children with fathers that have academic studies have higher odds of a higher rating for the gum health status by 1.786 times (95% C.I. = 1.228–2.598, *p* = 0.002); having a higher dental hygiene frequency increases the odds of a higher rating for all hygiene categories, consuming biscuits, cakes, cream cakes, sweet pies, etc., once/day or several times/day increased the odds of a lower rating while consumption of soft drinks increased the odds of a lower rating for weekly consumption or higher consumption categories.

### 3.3. Parents’ Perception of Their Children’s Dental Hygiene Habits

A total of 47.2% of the participating parents reported that their children brushed their teeth once daily, followed by brushing twice or more times a day (40.7%). Most of the children used a toothbrush (98.9%) and toothpaste (99%) for their dental hygiene, and only 22.4% included dental floss in their routine. The toothpaste contained fluoride in 64.3% of the cases. However, the use of fluoride toothpaste differed significantly based on living environments, predominantly among children living in an urban area (*p* = 0.001, Fisher’s Exact Test, Cramer’s V = 0.084, N = 1549). Results showed that use of fluoride toothpaste was significantly different according to parents’ education, as fathers (*p* = 0.001, Fisher’s Exact Test, Cramer’s V = 0.091, N = 1428) or mothers (*p* < 0.001, Fisher’s Exact Test, Cramer’s V = 0.127, N = 1509) with academic studies were significantly more likely to provide fluoride toothpaste to their children. Oppositely, children of parents without academic studies were more likely to not use fluoride toothpaste. The presence of fluoridated toothpaste did not show any statistically significant correlations with parents’ perception of their children’s dental and gum health status (*p* = 0.062, Cramer’s V = 0.078, N = 1487 and *p* = 0.572, Cramer’s V = 0.044, N = 1499 respectively, Fisher’s Exact Test).

Children who never brushed their teeth or brushed their teeth just a few times a month or a week were more likely to be associated with poor ratings of their dental and gum health status, as opposed to children who brushed their teeth daily, who were associated with average ratings of their dental health status and good ratings of their gum health status. Furthermore, children who brushed their teeth twice or more times a day were more associated with very good or excellent ratings of their dental health status (*p* < 0.001, Fisher’s Exact Test, Cramer’s V = 0.154, N = 1792).

### 3.4. Parents’ Perception of Their Children’s Eating and Smoking Habits

Regarding eating habits, parents reported 40.5% of children ate fruits once a day, 36.4% ate biscuits, cakes, cream cakes, sweet pies, etc., once a day, 30.5% ate jam or honey a few times a week, while for tea with sugar/honey, 26.7% consumed it a few times a month, and 25.9% consumed it a few times a week. The majority of children never consumed coffee with sugar/honey (76.9%) and 34.9% never had milk with sugar/honey. Children who consumed soft drinks, chewing gum with sugar and candy a few times a week represented 28%, 29%, and 32.1%, respectively. Child smoking was extremely rare, reported by only 4 parents (0.2%); due to this low frequency, smoking was excluded from inferential testing.

**Figure 1:**
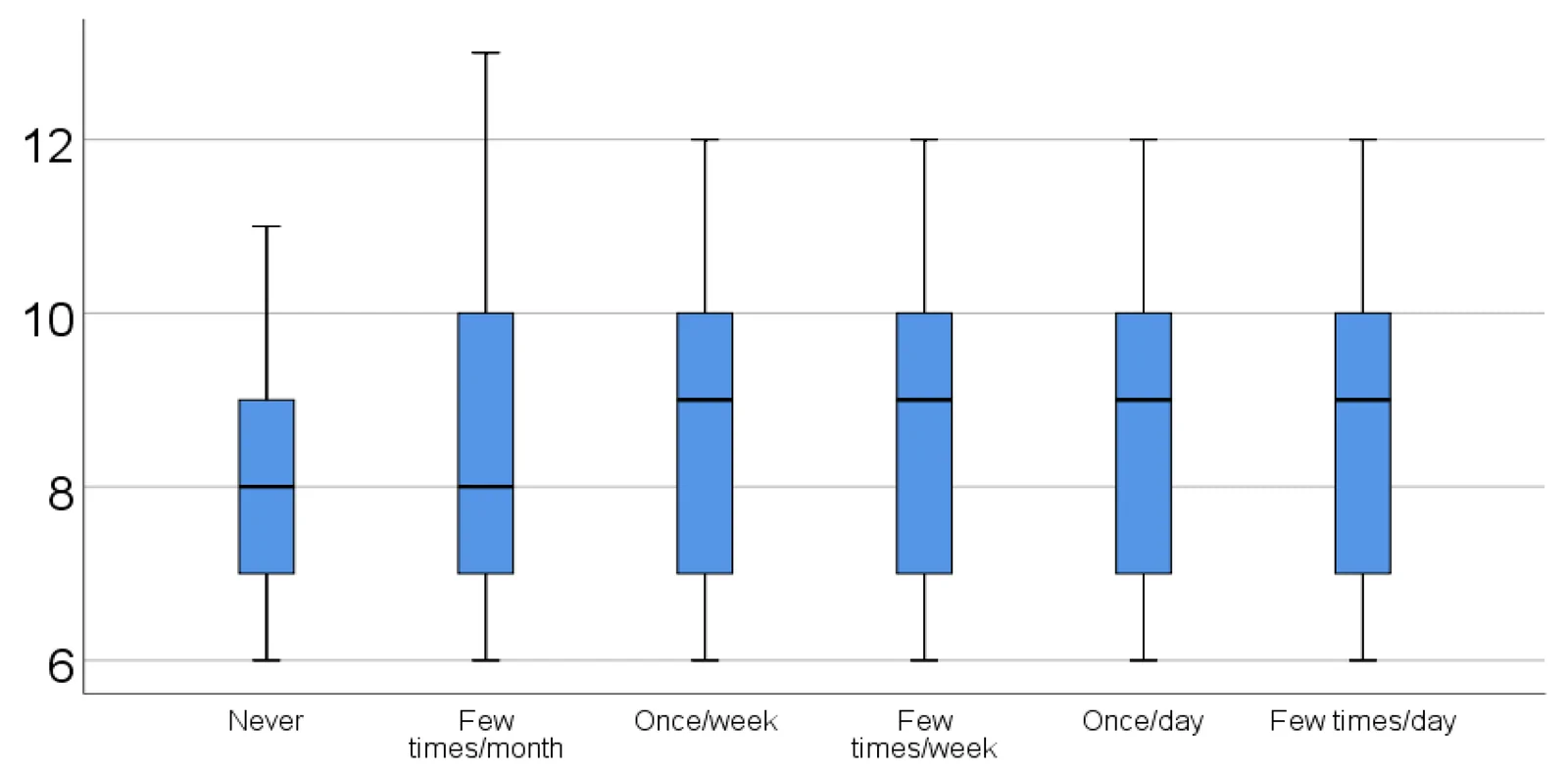
Comparison of participants’ age (years) according to the frequency of soft drinks consumption. Statistical significance *p* = 0.001, Kruskal–Wallis H Test.

Consumption of soft drinks increased with age in children, as illustrated in Figure 1 (*p* = 0.001, η^2^ = 0.008, Kruskal–Wallis H Test). Post hoc Dunn–Bonferroni comparisons showed that children who never consumed soft drinks had a statistically significant lower age (mean = 8.06 ± 1.38, 95% C.I. = 7.85–8.27, median = 8, IQR = 7–9) compared to children consuming soft drinks once a week (mean = 8.57 ± 1.41, 95% C.I. = 8.39–8.75, median = 9, IQR = 7–10; *p* = 0.009), a few times a week (mean = 8.52 ± 1.51, 95% C.I. = 8.39–8.65, median = 9, IQR = 7–10; *p* = 0.007), once a day (mean = 8.7 ± 1.53, 95% C.I. = 8.50–8.90, median = 9, IQR = 7–10; *p* < 0.001), or a few times a day (mean = 8.6 ± 1.52, 95% C.I. = 8.38–8.82, median = 9, IQR = 7–10; *p* = 0.014).

A GEE model with ordinal logistic structure that accounts for children classes clustering random effect shows that each increase in one year of children’s age increases the odds of being in a higher soft drink consumption category by 1.113 times (95% C.I. = 1.053–1.177, *p* < 0.001, Wald χ^2^(1) = 14.375, *p* < 0.001).

### 3.5. Parents’ Perception of Their Children’s Dental Pain

A percentage of 42.4% of the parents reported that their children had rarely had dental pain in the last 12 months; no significant differences regarding parents’ perception of their children’s dental pain were shown by distribution according to living environment (*p* = 0.06, Cramer’s V = 0.064, N = 1830, Fisher’s Exact Test). Parent-reported frequency of dental pain showed a significant relationship to parental ratings of dental and gum health status, (*p* < 0.001, Cramer’s V = 0.261, N = 1762/*p* < 0.001, Cramer’s V = 0.172, N = 1771, Fisher’s Exact Test). Parents who reported that their children never had dental pain in the last 12 months were more likely to rate their children’s dental health status as very good and excellent; similar results were reported for gum health status. Parents who reported that their children rarely had dental pain were more likely to rate their children’s dental and gum health status as good as opposed to excellent ratings. Parents who reported that their children occasionally had dental pain were more likely to give poor, average or good ratings of their children’s dental health status as opposed to very good or excellent ratings. Parents who reported that their children occasionally had dental pain were more likely to give poor, average, and good ratings of their children’s gums health status as opposed to excellent ratings. Parents who reported that their children had frequent dental pain were more likely to rate their dental and gum health status as poor as opposed to other ratings.

The relationship between dietary habits and the frequency of dental pain is presented in Table 2. A statistically significant association was observed between the consumption of biscuits, cakes, cream cakes, sweet pies, etc., or soft drinks and parent-reported dental pain frequency. Children who received biscuits, cakes, cream cakes, sweet pies, etc., a few times a week were more likely to never have had dental pain in the last 12 months, while children who received biscuits, cakes, cream cakes, sweet pies, etc., a few times a day had often experienced dental pain in the last 12 months (*p* < 0.001, Fisher’s Exact Test). Similarly, children who never received soft drinks or just received such products a few times a month were more likely to never have had dental pain in the last 12 months, while children who received soft drinks once or more than once a day had often experienced dental pain during the last 12 months (*p* < 0.001, Fisher’s Exact Test).

Prediction of dental pain frequency in a GEE model, using predictors which could impact dental health aspects (child age, gender, environment, academic studies for the father/mother, frequency of dental hygiene, usage of fluoride toothpaste and frequency of biscuits, cakes, cream cakes, sweet pies, etc., or soft drinks) reveals that sons had an increased chance for a lower pain category by 1.204 times (95% C.I. = 1.015–1.428, *p* = 0.033); dental hygiene practiced at least once/day or more times/day determined an increase chance for a lower pain category, consumption of soft drinks increased the odds of a higher pain rating for weekly consumption or higher consumption categories, while consumption of lower frequency categories in cases of biscuits, cakes, cream cakes, sweet pies, etc., (few times/week or once/week) increased the odds of a lower pain rating.

### 3.6. Parents’ Perception of Their Children’s Dental Visits

During the last 12 months, 21.4% of the children had only one dental visit, either for routine controls (38.5%) treatment (24.5%) or due to existing dental pain (37%), while 20% of the children never had a dental visit in their lifetime. Of the 369 parents reporting that their children never visited a dentist, only 93 parents provided a reason for absence of dental visits, and among these parents, the most quoted reason was parent-perceived lack of need for such services (76.3%), followed by treatment costs (18.3%).

Frequency of dental visits during the last 12 months differed significantly between rural and urban areas. Children who never visited a dentist in their lifetime were significantly more likely to be living in a rural environment, while participants who had visited the dentist two to four times during the last 12 months were significantly more likely to be living in an urban area (*p* < 0.001, Cramer’s V = 0.149, N = 1849, Fisher’s Exact Test). The frequency of dental visits did not show any statistically significant differences regarding the children’s gender (*p* = 0.414, Cramer’s V = 0.057, N = 1849, Fisher’s Exact Test), median age (*p* = 0.282, η^2^ = 0.001, N = 1849, Kruskal–Wallis H Test), dental health status (*p* = 0.086, Cramer’s V = 0.071, N = 1773, Fisher’s Exact Test), or gum health status (*p* = 0.062, Cramer’s V = 0.072, N = 1783, Fisher’s Exact Test). The frequency of dental visits did not show statistically significant differences between children regarding their satisfaction with their dental appearance (*p* = 0.352, Cramer’s V = 0.064, N = 1604, Fisher’s Exact Test), smile avoidance (*p* = 0.636, Cramer’s V = 0.052, N = 1713, Fisher’s Exact Test) or existence of social issues (*p* = 0.403, Cramer’s V = 0.064, N = 1644, Fisher’s Exact Test).

Parents’ academic background was another factor significantly correlated with the frequency of children’s dental visits. Children whose parents had academic studies were more likely to have had four dental visits during the last year, as opposed to never having a dental visit or having fewer dental visits in the last 12 months (*p* < 0.001, Cramer’s V = 0.207, N = 1703/*p* < 0.001, Cramer’s V = 0.300, N = 1797, Fisher’s Exact Test). Parents without academic studies were more likely to have never taken their children for a dental visit or have never or only once taken their children for a dental visit during the last 12 months.

The relationship between the frequency of dental visits and dental pain is presented in Table 2. A statistically significant association was observed between the frequency of dental visits and parent-reported occurrence of dental pain, as parents of children who had never had a dental visit in their lifetime were more likely to report that their children were not experiencing dental pain (*p* < 0.001, Fisher’s Exact Test). Conversely, higher dental visits frequencies were associated with frequent occurrence of dental pain, as children who had more than three dental visits in the last 12 months were more likely to have experienced often dental pain episodes. Moreover, the reason for the children’s most recent dental visit was significantly associated with parent-reported frequency of dental pain, as shown in Table 2, while routine dental check-ups were predominantly associated with the absence of dental pain in the last year (*p* < 0.001, Fisher’s Exact Test).

Frequency of dental hygiene was associated with the reason for dental visits, while the presence of fluoride toothpaste did not show any statistically significant relationship with the reason for the last dental visit (*p* = 0.750, Fisher’s Exact Test); the result is detailed in Table 3. Children who brushed their teeth a few times per week were more likely to attend the dental office for reasons associated with dental pain. Children who brushed their teeth once a day were more likely to have a dental visit for dental pain, rather than routine check-up. Children who brushed their teeth twice or more times a day were more likely to have dental visits for routine check-ups or dental treatments, rather than for dental pain.

Dietary habits were associated with the frequency of dental visits and the reason for the last dental visit, and the results are presented in Table 3. Children who seldom consumed soft drinks (a few times a month) were more likely to have had four dental visits in the last 12 months, while children who consumed soft drinks daily were more likely to never have had a dental visit (*p* < 0.001, Cramer’s V = 0.098, N = 1849, Fisher’s Exact Test). Frequency of soft drinks consumption was also significantly associated with the reason for the last dental visit, as children who never consumed soft drinks were more associated with routine check-ups, while children who consumed soft drinks once or a few times a day were more associated with dental visits due to dental pain (*p* < 0.001, Fisher’s Exact Test). Consumption of biscuits, cakes, cream cakes, sweet pies, etc., was not significantly associated with the reason for dental visits (*p* = 0.076, Fisher’s Exact Test).

Prediction of dental visit frequency in a GEE model, using predictors which could impact dental health aspects (child age, gender, environment, academic studies for the father/mother, usage of fluoride toothpaste and frequency of biscuits, cakes, cream cakes, sweet pies, etc., or soft drinks) reveals that children with fathers/mothers that have academic studies have an increased chance for a higher dental visit category by 1.168 times (95% C.I. = 1.002–1.361, *p* = 0.048)/2.223 times (95% C.I. = 1.914–2.583, *p* < 0.001); children who use fluoride toothpaste have an increased chance for a higher dental visit category by 1.324 times (95% C.I. = 1.141–1.536, *p* < 0.001).

Prediction of dental visit reason in a GEE model, using predictors which could impact dental health aspects (child age, gender, environment, academic studies for the father/mother, frequency of dental hygiene, usage of fluoride toothpaste and frequency of soft drinks) reveals that each decrease in patients’ age by one year increased the chance toward dental pain by 1.149 times (95% C.I.= 1.046–1.264, *p* = 0.004); children with mothers who have academic studies have a decreased chance toward dental pain by 1.466 times (95% C.I. = 1.045–2.053, *p* = 0.026) and an increased chance toward treatment by 1.443 times (95% C.I. = 1.001–2.082, *p* = 0.049); dental hygiene practiced two or more times/day decreased the chance toward dental pain by 3.846 times (95% C.I. = 1.106–13.333, *p* = 0.034), while consumption of soft drinks had an effect in decreasing the chance of having a routine check-up, while increasing the chance of having dental pain as the dental visit reason for all consumption categories.

### 3.7. Parents’ Perception of Their Children’s Oral Health Impacts

**Figure 2:**
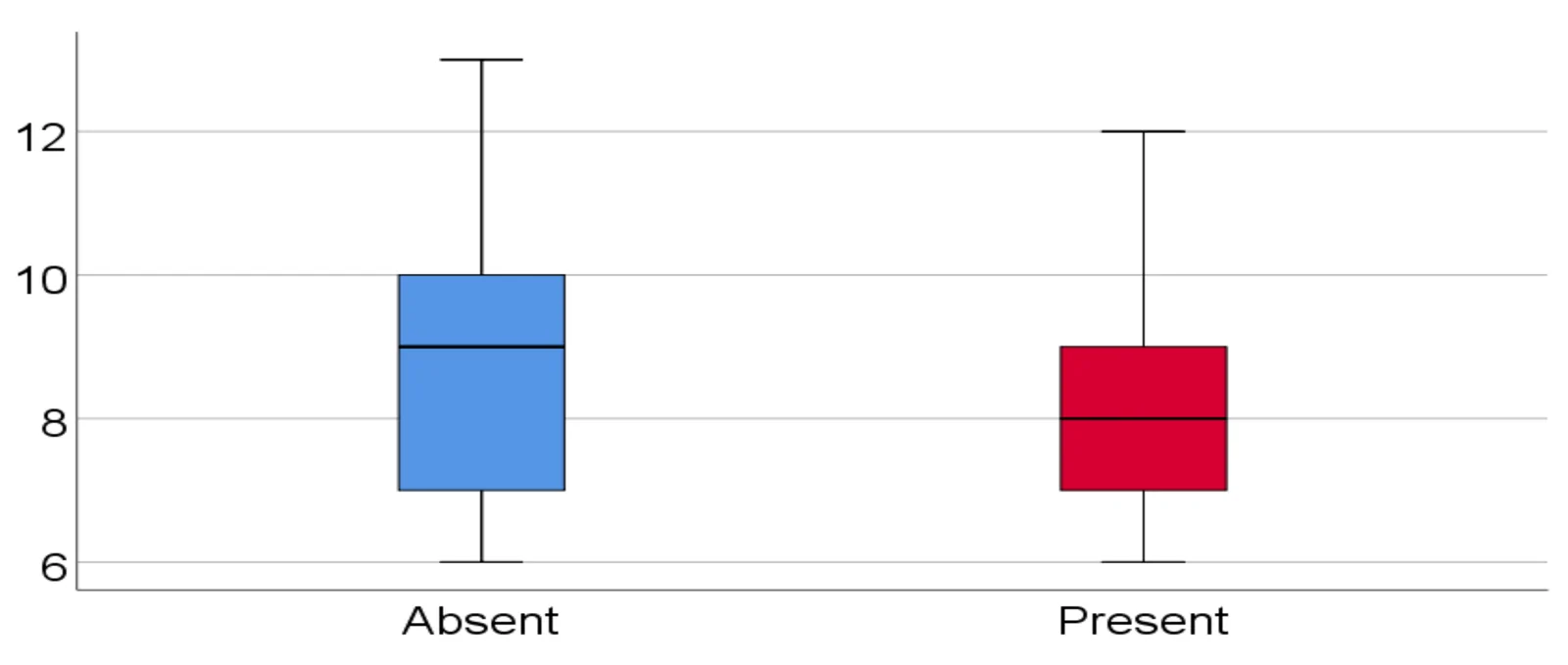
Comparison of participants’ age (years) according to the existence of difficulties while chewing food with hard consistency. Statistical significance *p* = 0.011, Mann–Whitney U Test.

Parents’ perception of their children’s oral health impacts showed that 26.1% considered their children to be dissatisfied with their current dental appearance. The invoked aspects related to oral health were difficulties in chewing hard food (19.7%) and school absenteeism due to dental problems (7.9%). The perceived oral health impacts showed no differences related to children’s gender (*p* > 0.05, Fisher’s Exact Test) or age (*p* > 0.05, Mann–Whitney U Test), except for significant differences in age regarding difficulties in chewing hard food (*p* = 0.011, r = 0.060, N = 1773, Mann–Whitney U Test), which was reported for younger children (mean age = 8.29 ± 1.52, 95% C.I. = 8.13–8.45, median = 8, IQR = 7–9) and was absent for older children (mean age = 8.5 ± 1.47, 95% C.I. = 8.43–8.58, median = 9, IQR = 7–10), as shown in Figure 2, along with a significantly higher age in children with smile avoidance (*p* = 0.033, r = 0.051, N = 1733, mean 95% C.I. = 8.51–9.11, median = 9, IQR = 8–10 vs. mean 95% C.I. = 8.39–8.53, median = 8, IQR = 7–10) or unsatisfaction toward their dental aspect (*p* = 0.001, r = 0.081, N = 1622, mean 95% C.I. = 8.55–8.83, median = 9, IQR = 7.98–10 vs. mean 95% C.I. = 8.33–8.5, median = 8, IQR = 7–10). Also, patients with chewing difficulties in hard food (22.4% vs. 16.8%, Cramer’s V = 0.070, N = 1773, *p* = 0.003) or food chewing difficulties in general (15.4% vs. 9.7%, Cramer’s V = 0.086, N = 1754, *p* < 0.001) were more frequently sons, rather than daughters.

A GEE model with binomial logistic structure that accounts for children classes clustering random effect shows that each decrease in one year of children’s age increases the odds of having difficulties while chewing food with hard consistency by 1.101 times (95% C.I. = 1.064–1.139, *p* < 0.001, Wald χ^2^(1) = 32.080, *p* < 0.001).

Parent-reported children’s dissatisfaction with their own dental appearance was associated with parent-perceived children’s dental health. Dissatisfaction was more frequent among children who had poor or average ratings of their dental and gum health status, while satisfaction was more frequently associated with excellent or very good ratings (*p* < 0.001, Cramer’s V = 0.388, N = 1570/*p* < 0.001, Cramer’s V = 0.297, N = 1580, Fisher’s Exact Test). Similarly, children who avoided smiling or laughing were more likely to receive lower ratings of their dental health, and were frequently associated with poor or average ratings of their dental and gum health status, while children who did not avoid smiling or laughing were associated with very good ratings of their dental health and excellent or very good ratings of their gum health status (*p* < 0.001, Cramer’s V = 0.147, N = 1671, and *p* = 0.001, Cramer’s V = 0.106, N = 1681 respectively, Fisher’s Exact Test). The reported presence or absence of social issues among children showed no significant statistical differences related to ratings of their dental and gum health status (*p* = 0.130, Cramer’s V = 0.066, N = 1605 and *p* = 0.290, Cramer’s V = 0.051, N = 1618 respectively, Fisher’s Exact Test). Unsurprisingly, children who avoided smiling or laughing and who had social issues related to oral health were significantly more likely to be dissatisfied with their own dental appearance (*p* < 0.001, Cramer’s V = 0.351, N = 1556/*p* < 0.001, Cramer’s V = 0.225, N = 1492, Fisher’s Exact Test).

Reported impacts related to oral health such as school absenteeism, difficulties with chewing food of any consistency or only of hard consistency were not associated with the use of toothpaste with fluoride content (*p* = 0.613, Cramer’s V = 0.015, N = 1481, *p* = 0.130, Cramer’s V = 0.040, N = 1482, and *p* = 0.506, Cramer’s V = 0.018, N = 1473 respectively, Fisher’s Exact Test). Furthermore, the analyzed oral health impacts were associated with the parent’s education level. Mothers with academic studies were more likely to perceive their children as being satisfied with their dental appearance, as not avoiding to smile or laugh or as not having social issues (*p* < 0.001, Cramer’s V = 0.158, N = 1585/*p* < 0.001, Cramer’s V = 0.119, N = 1691/*p* < 0.001, Cramer’s V = 0.096, N = 1626, Fisher’s Exact Test). Similarly, fathers with academic studies were more likely to perceive their children as being satisfied with their dental appearance, as not avoiding to smile or laugh or as not having social issues (*p* < 0.001, Cramer’s V = 0.129, N = 1496, *p* = 0.007, Cramer’s V = 0.067, N = 1602, and *p* = 0.03, Cramer’s V = 0.057, N = 1534, respectively, Fisher’s Exact Test).

The frequency of consumption of cariogenic food and beverages was significantly associated with school absenteeism and difficulties in mastication. Differences in the distribution of school absenteeism or difficulties in food chewing across consumption categories were assessed using Z-tests with Bonferroni correction, indicating that these issues significantly depended on food consumption, as detailed in Table S2. Frequency of consumption of fresh fruit showed no association with school absenteeism or with perceived difficulties in chewing in general (*p* = 0.128, and *p* = 0.192, respectively, Fisher’s Exact Test), but was significantly associated with lesser difficulties in chewing hard foods (*p* = 0.007, Fisher’s Exact Test).

The GEE models used in the manuscript are detailed in Table S3.

## 4. Discussion

This cross-sectional study, conducted within a school-based oral health education program, evaluated parent-reported oral health status, oral hygiene habits, and dietary patterns in primary schoolchildren. Families that were characterized by a more consistent home-based prevention, which included regular toothbrushing, fluoride toothpaste use and less intake of cariogenic foods, were associated with a higher tendency to seek dental care regularly for routine check-ups. Contrarily, families that accessed dental services mainly in response to symptoms were characterized by poorer dietary and hygiene-related habits, being influenced by social and educational background. While routine check-ups were the single most common individual reason for dental visits (38.5%), the majority of visits overall (61.5%) were non-preventive. Dental attendance was interpreted as symptom-driven and non-preventive, if parents reported either active dental treatment (24.5%) or pain (37%) as the primary reason for the child’s dental visit. The existence of symptoms related to oral health problems represents a factor associated with dental service seeking, which aligns with wider evidence that problem-oriented or delayed care-seeking in children is linked to contextual factors, including social disadvantage and uneven access to preventive services [15,16,17]. The association between pain frequency, pain-driven dental visits, and less favorable parent-perceived oral health suggests that for many families, dental care is sought mainly when problems become noticeable and cannot be avoided anymore, which points to unmet needs and delayed care, rather than preventive dental visits related to routine check-ups. By linking dental service utilization with pain, this behavior potentially influences long-term negative consequences upon children’s and future adults’ perceptions and attitudes toward oral care and oral health [18]. Consequently, a potential clinical and educational implication of our findings is that clinical management must prioritize painless care delivery using non-pharmacological behavioral guidance [19], alongside advanced local anesthesia methods [20,21] and minimally invasive operative procedures [22], which are in alignment with the objectives of the Erasmus+ PaFein+ project [23]. Integrating these techniques into routine practice is crucial to changing family perceptions of the dental setting, transforming potentially traumatic interventions into stress-free experiences that encourage timely, preventive dental service utilization [24].

Concurrently, the results indicate that prevention is more common in families with higher parental education and living in urban residences. Higher parental education was consistently associated with more frequent dental visits and use of fluoride toothpaste. It was also associated with differences in parents’ perception of the children’s dental health and reported psychosocial impact. Importantly, these outcomes reflect parental perceptions and reports, rather than clinically verified oral health status, which would require objective professional dental examination. Our results are consistent with those of Perpelea et al., who administered the same WHO-based questionnaire and reported that the education level of the adults living with the child was associated with parental perceptions of dental and gingival health, oral hygiene practices and their frequency, and the use of fluoride toothpaste and brushing aids [14]. Similar education-linked patterns have been reported in other population-based or school-linked assessments, where parental education, health literacy, and socioeconomic context were related to both preventive behaviors and dental service use [16,25]. These clinical and educational priorities mirror the strategic objectives of the WHO Global Strategy and Action Plan on Oral Health for 2023–2030 [26], which emphasizes reorienting oral health services from reactive care toward primary prevention, health promotion and the integration of patient-centered care models.

The association between frequent intake of cariogenic foods and beverages and dental pain is consistent with the established evidence regarding the link between sugar and dental caries with its subsequent symptoms [27,28,29]. A similar co-occurrence of cariogenic dietary exposure and substantial caries experience has been reported by Jiménez-Núñez et al., who found frequent consumption of sugar-containing beverages and sweets (with urban children reporting higher soft drink intake and rural children more often consuming sugary drinks before bedtime) alongside a high caries burden measured clinically by DMFT index, particularly in rural areas [4]. Importantly, the positive relationship observed in our study between age and consumption of soft drinks is in accordance with findings from other research groups showing that cariogenic beverage choices intensify with age and autonomy, reinforcing the case for early prevention strategies among parents and school staff [30].

The results linking cariogenic intake to school absenteeism are particularly relevant from a public health perspective [31]. Although the etiology of school absenteeism is multifactorial and cannot be solely attributed to consequences of oral conditions in a cross-sectional design based on parents’ perceptions, the consistent sugar exposure might be incriminated as a factor that is able to increase dental caries burden and pain, and subsequently daily functioning and school participation [32]. Our findings are compatible with the other studies reporting that school absenteeism is more consistently linked to symptoms and unmet needs than to clinically measured caries alone. In their review, Ruff et al. reported mixed evidence for associations between objectively assessed caries indices (e.g., DMFT/severe caries) and absenteeism, with several adjusted analyses showing no significant links, whereas subjectively reported dental pain and unmet oral health care needs were more consistently associated with missed school days [8]. Additional evidence on the burden of oral conditions supports the interpretation that oral symptoms can translate into meaningful limitations in daily functioning and quality of life. A systematic review concluded that common oral conditions, including dental caries, are generally associated with poorer health-related quality of life, reflecting impacts that extend beyond clinical findings [33].

In our findings, parent-reported chewing difficulties were associated with higher cariogenic food intake and symptom-related outcomes. At the same time, the association between more frequent cariogenic intake and chewing difficulties may be bidirectional: a cariogenic diet can increase caries experience and dental pain, subsequently impairing chewing ability, while chewing discomfort may also encourage the preference for softer, easier-to-tolerate meals that may often be cariogenic. Similar bidirectional interpretations have been discussed in other studies examining dietary patterns, oral symptoms, and functional limitations, particularly in cross-sectional designs [27,29]. Notably, because our findings rely on parents’ perceptions and the study is cross-sectional, we cannot establish causality. Nonetheless, the results support integrated parent education that links diet, early symptom recognition, routine preventive dental visits, rather than addressing these topics in isolation.

The reason for dental visits, routine dental check-ups versus pain-driven dental service utilization, is suggestive for a family’s preventive orientation. Home-based behaviors, like parent-reported toothbrushing frequency and use of fluoride toothpaste, are associated with patterns of dental visit frequency and with consequences of dental caries as reported by parents, such as pain, chewing difficulties, and school absenteeism. This interpretation is consistent with studies showing that early routine dental visits are of utmost importance, and that social inequalities influence whether families seek preventive care or delay dental visits until symptoms appear [15].

The outcomes analyzed in this study are parent-reported perceptions and behaviors, and not the direct result of children-reported data or outcomes of clinical examinations; this might induce reporting bias. Considering the cross-sectional design of the study, the associations between the analyzed variables should be interpreted with caution. Confounding by socioeconomic factors, access to care, and health literacy is plausible, particularly in analyses involving environment and parental education. Because the survey was anonymous and no personal identifiers were collected, duplicate submissions could not be checked, and it was not possible to determine whether the same parent completed questionnaires for more than one child enrolled in the program or how a target child was selected when more than one sibling was eligible. The sampling process may have introduced selection bias at both the teacher/class and parent-response levels, limiting the generalizability of the findings beyond the achieved study sample. Several questionnaire items included explicit “I do not know/I cannot remember/estimate” options. These responses were treated as non-informative and excluded item-wise, leaving complete cases for pairwise and multivariable analyses. Although this prevented misclassifying uncertain answers as substantive responses, it introduces potential item-nonresponse bias if parents who were unable to remember or estimate differed systematically from those who provided definitive answers. Furthermore, item-wise exclusion modestly reduced sample sizes for affected variables. These limitations highlight the need for integrating these results within broader studies with longitudinal designs and should prioritize clinical validation to reduce reliance on perceptions and to clarify directionality.

## 5. Conclusions

Our study presents results emerging from online responses given by parents participating in a school-based oral health program, demonstrating that despite favorable parental perceptions of schoolchildren’s oral health, dental pain is still reported, alongside functional and psychosocial impacts of oral diseases. Dietary findings also indicate that a more frequent cariogenic intake was associated with dental pain, chewing difficulties, and school absenteeism, supporting focused messaging on reducing frequent sugary snacks and soft drinks, especially as children get older. Moreover, dental visits are predominantly reported following symptom occurrence, associated with treatments or dental pain, rather than routine prevention. Socioeconomic and environmental factors are related to parent-perceived oral health and care-seeking behaviors, as lower parental education and rural residence were associated with less favorable outcomes, indicating priority targets for parent-focused education and outreach. Establishing effective preventive approaches will require equity-focused oral health education that addresses oral hygiene measures, cariogenic dietary habits and promotes routine dental visits, specifically targeting rural communities and parents with lower educational backgrounds.

## Figures and Tables

**Table 1:** Distribution of oral health-related parameters according to gender and living environment (responses to questions Q5–Q15).

**Q5. Current Dental Health (Nr., %) (N = 1792)**		**Poor**			**Average**				**Good**				**Very Good**				**Excellent**			***p*-Value**
		**106 (5.9%)**			**217 (12.1%)**				**908 (50.7%)**				**429 (23.9%)**				**132 (7.4%)**			
Daughter	Nr.	46			87 *				439				222				77 *			0.007 Cramer’s V = 0.089 **
	%	5.3%			10% *				50.4%				25.5%				8.8% *			
Son	Nr.	60			130 *				469				207				55 *			
	%	6.5%			14.1% *				50.9%				22.5%				6% *			
Q5. Current gums health (Nr., %) (N = 1803)		**Poor**			**Average**				**Good**				**Very Good**				**Excellent**			
		29 (1.6%)			114 (6.3%)				749 (41.5%)				627 (34.8%)				284 (15.8%)			
Daughter	Nr.	11			46				339 *				313				165 *			**<0.001** **Cramer’s V = 0.101**
	%	1.3%			5.3%				38.8% *				35.8%				18.9% *			
Son	Nr.	18			68				410 *				314				119 *			
	%	1.9%			7.3%				44.1% *				33.8%				12.8% *			
Q6. Dental pain in the last 12 months (Nr., %) (N = 1830)		**Never**				**Rarely**					**Occasionally**					**Often**				
		541 (29.6%)				776 (42.4%)					413 (22.6%)					100 (5.5%)				
Rural	Nr.	241				375					193					59				***p* = 0.060** **Cramer’s V = 0.064**
	%	27.8%				43.2%					22.2%					6.8%				
Urban	Nr.	300				401					220					41				
	%	31.2%				41.7%					22.9%					4.3%				
Q7. Dental visit in the last 12 months (Nr., %) (N = 1849)		**Never visited a dentist**	**Never visited a dentist in the last 12 months**				**Once**			**Twice**		**Three times**			**Four times**				**Five times or more**	
		369 (20%)	216 (11.7%)				395 (21.4%)			323 (17.5%)		202 (10.9%)			135 (7.3%)				209 (11.3%)	
Daughter	Nr.	178	99				183			166		110			68				96	***p* = 0.414** **Cramer’s V = 0.057**
	%	19.8%	11%				20.3%			18.4%		12.2%			7.6%				10.7%	
Son	Nr.	191	117				212			157		92			67				113	
	%	20.1%	12.3%				22.3%			16.5%		9.7%			7.1%				11.9%	
Rural	Nr.	223 *	114				176			134 *		87			50 *				97	**<0.001** **Cramer’s V = 0.149**
	%	25.3% *	12.9%				20%			15.2% *		9.9%			5.7% *				11%	
Urban	Nr.	146 *	102				219			189 *		115			85 *				112	
	%	15.1% *	10.5%				22.6%			19.5% *		11.9%			8.8% *				11.6%	
Q8. Last dental visit—Reason (Nr., %) (N = 1250)		**Routine check-up**						**Treatment**						**Dental pain**						
		481 (38.5%)						306 (24.5%)						463 (37%)						
Q8. Reason for absence of dental visit (Nr., %) (N = 93)		**No need of medical services**				**Low financial possibilities**					**Transportation issues**					**Long waiting lists**				
		71 (76.3%)				17 (18.3%)					3 (3.2%)					2 (2.2%)				
Q9. Frequency—Dental hygiene (Nr., %)		**Never**		**Few times/month**				**Once/week**			**Few times/week**			**Once/day**				**Twice or more/day**		
		6 (0.3%)		30 (1.6%)				33 (1.8%)			157 (8.4%)			883 (47.2%)				761 (40.7%)		
Q10-Q12. Use of dental products (Nr., %)		**Toothbrush**	**Wooden toothpicks**				**Plastic toothpicks**			**Dental floss**		**Other**			**Toothpaste**				**Fluoride toothpaste (N = 1549)**	
		1850 (98.9%)	335 (17.9%)				125 (6.7%)			418 (22.4%)		227 (12.1%)			1852 (99%)				996 (64.3%)	
Q13. Oral health impacts (Nr., %)		**Unsatisfying dental aspect (N = 1622)**		**Smile/laugh avoidance (N = 1733)**				**Social issues (N = 1664)**			**School class avoidance (N = 1776)**			**Difficult consumption of food with hard consistency (N = 1773)**				**Difficulties while chewing food (N = 1754)**		
		424 (26.1%)		98 (5.7%)				37 (2.2%)			141 (7.9%)			349 (19.7%)				222 (12.7%)		
Daughter	Nr.	221		46				19			59			144				83		
	%	27.4%		5.5%				2.3%			6.9%			16.8%				9.7%		
Son	Nr.	203		52				18			82			205				139		
	%	24.9%		5.8%				2.1%			9%			22.4%				15.4%		
*p* *		0.258 Cramer’s V = 0.029		0.756 Cramer’s V = 0.009				0.868 Cramer’s V = 0.007			0.114 Cramer’s V = 0.039			**0.003** **Cramer’s V = 0.070**				**<0.001** **Cramer’s V = 0.086**		
Q14. Food consumption (Nr., %)		**Never**		**Few times/month**				**Once/week**			**Few times/week**			**Once/day**				**Few times/day**		
Fresh fruit		22 (1.2%)		38 (2%)				57 (3%)			488 (26.1%)			**758 (40.5%)**				507 (27.1%)		
Biscuits, cakes, cream cakes, sweet pies, etc		6 (0.3%)		130 (7%)				159 (8.5%)			614 (32.8%)			**680 (36.4%)**				281 (15%)		
Jam/honey		332 (17.8%)		450 (24.1%)				225 (12%)			**570 (30.5%)**			256 (13.7%)				37 (2%)		
Soft drinks		169 (9%)		**529 (28.3%)**				239 (12.8%)			**523 (28%)**			224 (12%)				186 (9.9%)		
Chewing gum with sugar		375 (20.1%)		**529 (28.3%)**				194 (10.4%)			**542 (29%)**			163 (8.7%)				67 (3.6%)		
Candy		109 (5.8%)		507 (27.1%)				260 (13.9%)			**600 (32.1%)**			262 (14%)				132 (7.1%)		
Milk with sugar/honey		**652 (34.9%)**		302 (16.1%)				169 (9%)			434 (23.2%)			231 (12.4%)				82 (4.4%)		
Tea with sugar/honey		456 (24.4%)		**500 (26.7%)**				181 (9.7%)			**485 (25.9%)**			189 (10.1%)				59 (3.2%)		
Coffee with sugar/honey		**1438 (76.9%)**		28 (1.5%)				31 (1.7%)			311 (16.6%)			53 (2.8%)				9 (0.5%)		
Q15. Smoking (Nr., %)		4 (0.2%)																		

* Fisher’s Exact Test, statistical significance (*p* < 0.05), ** Not significant after Holm–Bonferroni adjustment.

**Table 2:** Distribution of the participants according to dental pain frequency and frequency of dental visits, reason for dental visit, and dietary habits.

	Dental Pain Frequency				
Dental visit (Nr., %) (N = 1813)	**Never**	**Rarely**	**Occasionally**	**Often**	***p*** *
Never visited a dentist	**159 (29.7%)**	**120 (15.6%)**	**63 (15.4%)**	**16 (16.2%)**	**<0.001** **Cramer’s V = 0.171**
Never visited a dentist in the last 12 months	**84 (15.7%)**	93 (12.1%)	**32 (7.8%)**	**4 (4%)**	
Once	**126 (23.6%)**	162 (21%)	82 (20%)	**11 (11.1%)**	
Twice	**77 (14.4%)**	**166 (21.6%)**	65 (15.9%)	12 (12.1%)	
Three times	**35 (6.5%)**	**95 (12.3%)**	**53 (13%)**	**17 (17.2%)**	
Four times	**18 (3.4%)**	**53 (6.9%)**	**51 (12.5%)**	11 (11.1%)	
Five times or more	**36 (6.7%)**	81 (10.5%)	**63 (15.4%)**	**28 (28.3%)**	
Reason for last dental visit (Nr., %) (N = 1229)					
Routine check-up	**215 (74.1%)**	**187 (34.1%)**	**62 (19.8%)**	**7 (9.1%)**	**<0.001** **Cramer’s V = 0.329**
Treatment	**52 (17.9%)**	**153 (27.9%)**	79 (25.2%)	18 (23.4%)	
Dental pain	**23 (7.9%)**	**209 (38.1%)**	**172 (55%)**	**52 (67.5%)**	
Biscuits, cakes, cream cakes, sweet pies, etc., consumption (Nr., %) (N = 1830)					
Never	2 (0.4%)	2 (0.3%)	1 (0.2%)	1 (1%)	**<0.001** **Cramer’s V = 0.093**
Few times/month	43 (7.9%)	45 (5.8%)	30 (7.3%)	8 (8%)	
Once/week	59 (10.9%)	63 (8.1%)	29 (7%)	5 (5%)	
Few times/week	**204 (37.7%)**	256 (33%)	**121 (29.3%)**	**23 (23%)**	
Once/day	182 (33.6%)	293 (37.8%)	154 (37.3%)	35 (35%)	
Few times/day	**51 (9.4%)**	**117 (15.1%)**	78 (18.9%)	**28 (28%)**	
Soft drinks consumption (Nr., %) (N = 1830)					
Never	**71 (13.1%)**	**56 (7.2%)**	**28 (6.8%)**	9 (9%)	**<0.001** **Cramer’s V = 0.116**
Few times/month	**180 (33.3%)**	**223 (28.7%)**	102 (24.7%)	**13 (13%)**	
Once/week	62 (11.5%)	108 (13.9%)	55 (13.3%)	10 (10%)	
Few times/week	137 (25.3%)	222 (28.6%)	133 (32.2%)	23 (23%)	
Once/day	**56 (10.4%)**	91 (11.7%)	50 (12.1%)	**20 (20%)**	
Few times/day	**35 (6.5%)**	**76 (9.8%)**	**45 (10.9%)**	**25 (25%)**	

* Fisher’s Exact Test, statistical significance (*p* < 0.05).

**Table 3:** Distribution of the participants according to the reason for the last dental visit and frequency of dental hygiene, fluoride toothpaste usage and dietary habits.

	Reason for the Last Dental Visit						
Dental hygiene (*N* = 1250)	**Check-up**		**Treatment**		**Dental pain**		*p* *
	**Nr.**	**%**	**Nr.**	**%**	**Nr.**	**%**	
Few times/month	3	0.6%	2	0.7%	10	2.2%	**<0.001** **Cramer’s V = 0.169**
Once/week	4	0.8%	4	1.3%	11	2.4%	
Few times/week	**20**	**4.2%**	**13**	**4.2%**	**54**	**11.7%**	
Once/day	**188**	**39.1%**	131	42.8%	**239**	**51.6%**	
Twice or more/day	**266**	**55.3%**	**156**	**51%**	**149**	**32.2%**	
Fluoride toothpaste usage (*N* = 1057)							
Absent	129	31.7%	80	30.7%	130	33.3%	0.750 Cramer’s V = 0.024
Present	278	68.3%	181	69.3%	259	66.7%	
Biscuits, cakes, cream cakes, sweet pies, etc., consumption (*N* = 1250)							
Never	1	0.2%	3	1%	2	0.4%	0.076 Cramer’s V = 0.082
Few times/month	30	6.2%	23	7.5%	36	7.8%	
Once/week	51	10.6%	26	8.5%	31	6.7%	
Few times/week	179	37.2%	95	31%	138	29.8%	
Once/day	158	32.8%	119	38.9%	184	39.7%	
Few times/day	62	12.9%	40	13.1%	72	15.6%	
Soft drinks consumption (*N* = 1250)							
Never	**73**	**15.2%**	**29**	**9.5%**	**22**	**4.8%**	**<0.001** **Cramer’s V = 0.152**
Few times/month	142	29.5%	115	37.6%	136	29.4%	
Once/week	61	12.7%	46	15%	68	14.7%	
Few times/week	128	26.6%	88	28.8%	127	27.4%	
Once/day	**49**	**10.2%**	**15**	**4.9%**	**59**	**12.7%**	
Few times/day	**28**	**5.8%**	**13**	**4.2%**	**51**	**11%**	

* Fisher’s Exact Test, statistical significance (*p* < 0.05).

## Data Availability

The data presented in this study is available upon request from the corresponding author.

## Supplementary Materials

The following supporting information can be downloaded at https://www.mdpi.com/article/10.3390/children13101342/s1, Table S1: Questionnaire items in the English and Romanian versions. Table S2: Distribution of the children according to the existence of school absenteeism and difficulties while chewing food in relationship to dietary habits. Table S3: GEE models used in the manuscript.
