# Supplementary material for: Parent-Reported Oral Health Status and Dental Care Habits in Primary Schoolchildren: A Cross-Sectional Study
Source: Children (Basel). 2026 Oct 2;13(10):1342. doi: 10.3390/children13101342 (PMC13703419; doi:10.3390/children13101342)
Supplement: Supplementary file 1 [file children-13-01342-s001.zip › children-4529769-supplementary/Table S1- Supplementary Material.pdf]

Table S1. Questionnaire items in the English and Romanian versions.

Items from the English Version	Items from the Romanian Version
**Questionnaire for Parents of Children Participating in the *Little Dentists* Programme**	**Chestionar pentru Părintii Copiilor Înscriși în Programul *Micii Stomatologi***
**Please answer the following questions regarding your son's/daughter's oral health. Mark the box corresponding to the appropriate answer for each question.**	**Vă rugăm să răspundeți la următoarele întrebări legate de sănătatea orală a fiului/fiicei dvs. Bifați în căsuța corespunzătoare răspunsul care se potrivește la fiecare întrebare.**
**1. The child you are answering for is your:**<br><br>a. Son<br>b. Daughter	**1. Răspundeți pentru:**<br><br>a. Fiu<br>b. Fiică
**2. Where does the child for whom you are answering live?**<br><br>a. Urban area<br>b. Rural area	**2. Unde locuiește copilul pentru care răspundeți?**<br><br>a. Mediul urban<br>b. Mediul rural
**3. Your child's age (in completed years): ____________________**	**3. Vârsta copilului dvs. (în ani împliniți) ____________________**
**4. Your child's school grade: ____________________**	**4. Clasa copilului dvs.: ________________________**
**5. How would you describe the health of your son's/daughter's teeth and gums?**<br><br>Teeth Gums<br>a. Excellent<br>b. Very good<br>c. Good<br>d. Average<br>e. Poor<br>f. I do not know / I cannot estimate	**5. Cum ați descrie starea de sănătate a dinților și gingiilor fiului/fiicei dvs.?**<br><br>Dinți Gingii<br>a. Excelentă<br>b. Foarte bună<br>c. Bună<br>d. Satisfăcătoare<br>e. Precară<br>f. Nu știu/nu pot estima
**6. During the last 12 months, how often has your son/daughter experienced dental pain or discomfort affecting the teeth?**<br><br>a. Often<br>b. Occasionally<br>c. Rarely<br>d. Never<br>e. I do not know / I cannot estimate	**6. Cât de des, în ultimele 12 luni, fiul/fiica dvs. a avut dureri dentare sau disconfort la nivelul dinților?**<br><br>a. Des<br>b. Ocazional<br>c. Rar<br>d. Niciodată<br>e. Nu știu/nu pot estima
Now, please answer a few questions about how your son/daughter cares for their teeth.	*Acum vă rugăm să răspundeți la cateva întrebări legate de modul în care fiul/fiica dvs. își îngrijește dinții.*
**7. How often has your son/daughter visited a dentist during the last 12 months? (Select one answer only.)**<br><br>a. Once	**7. Cât de des a fost fiul/fiica dvs. la medicul stomatolog în ultimele 12 luni? (alegeți un singur răspuns)**<br><br>a. O dată

Items from the English Version	Items from the Romanian Version
b. Twice<br>c. Three times<br>d. Four times<br>e. Five times or more<br>f. Never visited a dentist in the last 12 months<br>g. Never visited a dentist<br>h. I do not know / I cannot remember	b. De două ori<br>c. De trei ori<br>d. De patru ori<br>e. De cinci ori sau mai mult<br>f. Niciodată în ultimele 12 luni<br>g. Niciodată<br>h. Nu știu/nu îmi amintesc
**8. What was the reason for the most recent dental visit? (Complete this question only if your child visited a dental practice during the last 12 months.)**<br><br>a. Dental pain or problems affecting the teeth, gums, or mouth<br>b. Treatment / multiple follow-up treatment visits<br>c. Routine check-up<br>d. I do not know / I cannot remember	**8. Care a fost motivul ultimei vizite la medicul stomatolog? (completați doar dacă în ultimele 12 luni a fost într-un cabinet stomatologic)**<br><br>a. Durere sau probleme la nivelul dinților, gingiilor, cavității orale<br>b. Tratament / vizite multiple<br>c. Control de rutină<br>d. Nu știu / nu îmi amintesc
**9. How often does your son/daughter brush their teeth?**<br><br>a. Never<br>b. Few times/month<br>c. Once/week<br>d. Few times/week (2–6 times)<br>e. Once/day<br>f. Twice or more/day	**9. Cât de des se spală pe dinți fiul/fiica dvs.?**<br><br>a. Niciodată<br>b. De câteva ori pe lună<br>c. O dată pe săptămână<br>d. De câteva ori pe săptămână (2-6 ori)<br>e. O dată pe zi<br>f. De două sau mai multe ori pe zi
**10. Which methods does your son/daughter use to clean their teeth or gums?**<br><br>Yes No<br>a. Toothbrush<br>b. Wooden toothpicks<br>c. Plastic toothpicks<br>d. Dental floss<br>e. Other (please specify): ____________________	**10. Care sunt metodele folosite de fiul/fiica dvs. pentru a-și curăța dinții sau gingiile?**<br><br>Da Nu<br>a. Periuță de dinți<br>b. Scobitori din lemn<br>c. Scobitori din plastic<br>d. Ață dentară<br>e. Altele (specificați): ____________________
**11. Does your son/daughter use toothpaste to brush their teeth?**<br><br>a. Yes<br>b. No	**11. Fiul/fiica dvs. folosește pastă de dinți pentru curățarea dinților?**<br><br>a. Da<br>b. Nu
**12. Does your son/daughter use fluoride toothpaste when brushing their teeth?**<br><br>a. Yes<br>b. No<br>c. I do not know	**12. Fiul/fiica dvs. folosește pastă de dinți cu fluor pentru periajul dentar?**<br><br>a. Da<br>b. Nu<br>c. Nu știu

Items from the English Version	Items from the Romanian Version
**13. Please indicate whether each of the following statements about your son's/daughter's oral health experience is true or false.**<br><br>True False I do not know<br>a. Is dissatisfied with the appearance of their teeth<br>b. Often avoids smiling or laughing because of the appearance of their teeth<br>c. Other children make fun of your son's/daughter's teeth<br>d. School absenteeism: dental pain or related discomfort has caused your son/daughter to miss one or more days of school<br>e. Your son/daughter has difficulties while chewing food with hard consistency<br>f. Your son/daughter has difficulties while chewing food	**13. Care din următoarele afirmații sunt adevărate/false în ceea ce privește experiența fiului/fiicei dvs. în legătură cu starea de sănătate orală?**<br><br>Adevărat Fals Nu știu<br>a. Nu este mulțumit de aspectul dinților<br>b. Adesea evită să zâmbească sau să râdă din cauza aspectului dinților<br>c. Alți copii se distrează pe seama dinților fiului/fiicei dvs.<br>d. Durerea de dinți sau disconfortul cauzat de aceasta au condus la lipsa de la cursuri la școală pentru una sau mai multe zile<br>e. Fiul/fiica dvs. are dificultăți atunci când mănâncă alimente dure<br>f. Fiul/fiica dvs. prezintă dificultăți în procesul de mestecare al alimentelor
**14. How often does your son/daughter eat or drink the following items, even in small amounts? Enter a number from 1 to 6 in each box, according to the scale below.**<br><br>6 = Few times/day 5 = Once/day 4 = Few times/week 3 = Once/week<br>2 = Few times/month 1 = Never<br><br>a. Fresh fruit<br>b. Biscuits, cakes, cream cakes, sweet pies, etc.<br>c. Jam or honey<br>d. Soft drinks<br>e. Chewing gum with sugar<br>f. Candy<br>g. Milk with sugar/honey<br>h. Tea with sugar/honey<br>i. Coffee with sugar/honey	**14. Cât de des mâncă sau bea fiul/fiica dvs. următoarele alimente, chiar și în cantități mici? Notați în fiecare căsuță o cifră de la 1-6, conform legendei de mai jos.**<br><br>6=De câteva ori pe zi 5=O dată pe zi 4=De câteva ori pe săptămână<br>3=O dată pe săptămână 2=De câteva ori pe lună 1=Niciodată<br><br>a. Fructe proaspete<br>b. Biscuiți, prăjituri, tort, plăcinte, etc.<br>c. Dulceață/miere<br>d. Băuturi răcoritoare acidulate sau neacidulate<br>e. Gumă de mestecat ce conține zahăr<br>f. Bomboane<br>g. Lapte cu zahăr/miere<br>h. Ceai cu zahăr/miere<br>i. Cafea cu zahăr/miere
**15. How often does your son/daughter smoke? (Select one answer only.)**<br><br>a. Every day<br>b. A few times a week<br>c. Once a week<br>d. A few times a month<br>e. Rarely<br>f. Never	**15. Cât de des fumează fiul/fiica dvs.? (Încercuiți o singură variantă de răspuns.)**<br><br>a. În fiecare zi<br>b. De câteva ori pe săptămână<br>c. O dată pe săptămână<br>d. De câteva ori pe lună<br>e. Rareori<br>f. Niciodată
**16. What is the highest level of education completed by the male adult with whom the child lives? (Select one answer only.)**<br><br>a. No formal schooling<br>b. Primary school not completed<br>c. Primary school<br>d. Lower secondary school<br>e. High school	**16. Ce nivel de educație a terminat adultul de sex masculin cu care copilul locuiește? (Încercuiți o singură variantă de răspuns.)**<br><br>a. Fără școală<br>b. Școala primară nefinalizată<br>c. Școală primară<br>d. Școală gimnazială<br>e. Liceu

Items from the English Version	Items from the Romanian Version
f. University education<br>g. Does not live with a male adult<br>h. I do not know	f. Studii universitare<br>g. Nu locuiește cu adult de sex masculin<br>h. Nu știu
**17. What is the highest level of education completed by the female adult with whom the child lives? (Select one answer only.)**<br><br>a. No formal schooling<br>b. Primary school not completed<br>c. Primary school<br>d. Lower secondary school<br>e. High school<br>f. University education<br>g. Does not live with a female adult<br>h. I do not know	**17. Ce nivel de educație a terminat adultul de sex feminin cu care copilul locuiește? (încercuiți o singură variantă de răspuns)**<br><br>a. Fără școală<br>b. Școala primară nefinalizată<br>c. Școală primară<br>d. Școală gimnazială<br>e. Liceu<br>f. Studii universitare<br>g. Nu locuiește cu adult de sex feminin<br>h. Nu știu
**Date of completion** **Town / City** **School**<br><br>Thank you!	**Data completării** **Oraș** **Școala**<br><br>Vă mulțumim!
