# Supplementary material for: Parent-Reported Oral Health Status and Dental Care Habits in Primary Schoolchildren: A Cross-Sectional Study
Source: Children (Basel). 2026 Oct 2;13(10):1342. doi: 10.3390/children13101342 (PMC13703419; doi:10.3390/children13101342)
Supplement: Supplementary file 1 [file children-13-01342-s001.zip › children-4529769-supplementary/Table S2- Supplementary Material.pdf]

Table S2. Distribution of the children according to the existence of school absenteeism and difficulties while chewing food in relationship to dietary habits.

Frequency of consumption		**School absenteeism**					**Difficulties while chewing food with hard consistency**					**Difficulties while chewing food**				
		NO		YES		p*	NO		YES		p*	NO		YES		p*
		**Nr.**	**%**	**Nr.**	**%**		**Nr.**	**%**	**Nr.**	**%**		**Nr.**	**%**	**Nr.**	**%**	
Fresh fruit (N=1776/ N=1773/ N=1754)	**Never**	20	1.2%	1	0.7%		13	0.9%	7	2%		14	0.9%	6	2.7%	
	**Few times/month**	35	2.1%	3	2.1%		**36**	**2.5%**	**1**	**0.3%**		35	2.3%	2	0.9%	
	**Once/week**	47	2.9%	8	5.7%	0.128, Cramer's V=0.069	**39**	**2.7%**	**17**	**4.9%**	**0.007* Cramer's V=0.091**	47	3.1%	6	2.7%	0.192 Cramer's V=0.068
	**Few times/week**	420	25.7%	34	24.1%		355	24.9%	94	26.9%		387	25.3%	62	27.9%	
	**Once/day**	679	41.5%	47	33.3%		593	41.6%	138	39.5%		636	41.5%	86	38.7%	
	**Few times/day**	434	26.5%	48	34%		388	27.2%	92	26.4%		413	27%	60	27%	
Biscuits, cakes, cream cakes, sweet pies, etc. (N=1776/ N=1773/ N=1754)	**Never**	6	0.4%	0	0%		5	0.4%	1	0.3%		5	0.3%	1	0.5%	
	**Few times/month**	118	7.2%	6	4.3%		99	7%	28	8%		108	7%	18	8.1%	
	**Once/week**	143	8.7%	10	7.1%	**0.006* Cramer's V=0.102**	133	9.3%	22	6.3%	**0.007* Cramer's V=0.095**	**143**	**9.3%**	**10**	**4.5%**	**0.009* Cramer's V=0.092**
	**Few times/week**	**551**	**33.7%**	**35**	**24.8%**		**486**	**34.1%**	**97**	**27.8%**		508	33.2%	68	30.6%	
	**Once/day**	586	35.8%	53	37.6%		509	35.7%	130	37.2%		561	36.6%	77	34.7%	
	**Few times/day**	**231**	**14.1%**	**37**	**26.2%**		**192**	**13.5%**	**71**	**20.3%**		**207**	**13.5%**	**48**	**21.6%**	
Jam or honey (N=1776/ N=1773/ N=1754)	**Never**	289	17.7%	30	21.3%		240	16.9%	73	20.9%		271	17.7%	36	16.2%	
	**Few times/month**	403	24.6%	35	24.8%		359	25.2%	77	22.1%		391	25.5%	47	21.2%	
	**Once/week**	199	12.2%	17	12.1%	**<0.001* Cramer's V=0.158**	178	12.5%	39	11.2%	**0.005* Cramer's V=0.103**	193	12.6%	25	11.3%	**<0.001* Cramer's V=0.129**
	**Few times/week**	**507**	**31%**	**23**	**16.3%**		445	31.3%	93	26.6%		463	30.2%	61	27.5%	
	**Once/day**	217	13.3%	25	17.7%		184	12.9%	53	15.2%		**195**	**12.7%**	**40**	**18%**	
	**Few times/day**	**20**	**1.2%**	**11**	**7.8%**		**18**	**1.3%**	**14**	**4%**		**19**	**1.2%**	**13**	**5.9%**	
Soft drinks (N=1776/ N=1773/ N=1754)	**Never**	**157**	**9.6%**	**4**	**2.8%**		139	9.8%	23	6.6%		140	9.1%	21	9.5%	
	**Few times/month**	**499**	**30.5%**	**18**	**12.8%**		**432**	**30.3%**	**84**	**24.1%**		**472**	**30.8%**	**42**	**18.9%**	
	**Once/week**	212	13%	22	15.6%	**<0.001* Cramer's V=0.191**	190	13.3%	39	11.2%	**<0.001* Cramer's V=0.182**	204	13.3%	24	10.8%	**<0.001* Cramer's V=0.148**
	**Few times/week**	453	27.7%	34	24.1%		406	28.5%	85	24.4%		426	27.8%	60	27%	
	**Once/day**	**179**	**10.9%**	**30**	**21.3%**		156	11%	49	14%		174	11.4%	34	15.3%	
	**Few times/day**	**136**	**8.3%**	**33**	**23.4%**		**101**	**7.1%**	**69**	**19.8%**		**116**	**7.6%**	**41**	**18.5%**	
Chewing gum with sugar (N=1776/ N=1773/ N=1754)	**Never**	**347**	**21.2%**	**13**	**9.2%**		300	21.1%	63	18.1%		324	21.1%	37	16.7%	
	**Few times/month**	486	29.7%	32	22.7%		422	29.6%	87	24.9%		**458**	**29.9%**	**48**	**21.6%**	
	**Once/week**	**166**	**10.2%**	**22**	**15.6%**	**<0.001* Cramer's V=0.150**	150	10.5%	37	10.6%	**0.001* Cramer's V=0.116**	158	10.3%	28	12.6%	**<0.001* Cramer's V=0.136**
	**Few times/week**	463	28.3%	43	30.5%		405	28.4%	101	28.9%		441	28.8%	62	27.9%	
	**Once/day**	131	8%	17	12.1%		112	7.9%	36	10.3%		**115**	**7.5%**	**31**	**14%**	
	**Few times/day**	**42**	**2.6%**	**14**	**9.9%**		**35**	**2.5%**	**25**	**7.2%**		**36**	**2.3%**	**16**	**7.2%**	
Candy (N=1776/ N=1773/ N=1754)	**Never**	99	6.1%	5	3.5%		87	6.1%	19	5.4%		91	5.9%	14	6.3%	
	**Few times/month**	**466**	**28.5%**	**24**	**17%**	**<0.001* Cramer's V=0.146**	408	28.7%	85	24.4%	**<0.001* Cramer's V=0.139**	**442**	**28.9%**	**50**	**22.5%**	**0.025* Cramer's V=0.089**
	**Once/week**	238	14.6%	19	13.5%		207	14.5%	44	12.6%		218	14.2%	32	14.4%	

	**Few times/week**	522	31.9%	42	29.8%		465	32.7%	97	27.8%		487	31.8%	68	30.6%	
	**Once/day**	215	13.1%	26	18.4%		181	12.7%	57	16.3%		207	13.5%	32	14.4%	
	**Few times/day**	**95**	**5.8%**	**25**	**17.7%**		**76**	**5.3%**	**47**	**13.5%**		**87**	**5.7%**	**26**	**11.7%**	
Milk with sugar/ honey (N=1776/ N=1773/ N=1754)	**Never**	**601**	**36.8%**	**31**	**22%**		512	36%	114	32.7%		**561**	**36.6%**	**63**	**28.4%**	
	**Few times/month**	277	16.9%	20	14.2%		249	17.5%	48	13.8%		264	17.2%	34	15.3%	
	**Once/week**	139	8.5%	18	12.8%	**<0.001***	133	9.3%	23	6.6%	**<0.001***	141	9.2%	12	5.4%	**<0.001***
	**Few times/week**	378	23.1%	30	21.3%	**Cramer's V=0.141**	335	23.5%	76	21.8%	**Cramer's V=0.145**	353	23%	49	22.1%	**Cramer's V=0.160**
	**Once/day**	**185**	**11.3%**	**27**	**19.1%**		**153**	**10.7%**	**57**	**16.3%**		**166**	**10.8%**	**40**	**18%**	
	**Few times/day**	**55**	**3.4%**	**15**	**10.6%**		**42**	**2.9%**	**31**	**8.9%**		**47**	**3.1%**	**24**	**10.8%**	
Tea with sugar/ honey (N=1776/ N=1773/ N=1754)	**Never**	411	25.1%	26	18.4%		362	25.4%	73	20.9%		389	25.4%	45	20.3%	
	**Few times/month**	**461**	**28.2%**	**28**	**19.9%**		394	27.7%	89	25.5%		**435**	**28.4%**	**47**	**21.2%**	
	**Once/week**	160	9.8%	14	9.9%	**<0.001***	139	9.8%	32	9.2%	**<0.001***	146	9.5%	23	10.4%	**<0.001***
	**Few times/week**	420	25.7%	27	19.1%	**Cramer's V=0.200**	382	26.8%	76	21.8%	**Cramer's V=0.154**	391	25.5%	51	23%	**Cramer's V=0.160**
	**Once/day**	**147**	**9%**	**28**	**19.9%**		**115**	**8.1%**	**55**	**15.8%**		**136**	**8.9%**	**36**	**16.2%**	
	**Few times/day**	**36**	**2.2%**	**18**	**12.8%**		**32**	**2.2%**	**24**	**6.9%**		**35**	**2.3%**	**20**	**9%**	
Coffee with sugar/ honey (N=1776/ N=1773/ N=1754)	**Never**	1284	78.5%	101	71.6%		1121	78.7%	258	73.9%		**1209**	**78.9%**	**162**	**73%**	
	**Few times/month**	25	1.5%	3	2.1%		23	1.6%	4	1.1%		23	1.5%	3	1.4%	
	**Once/week**	**22**	**1.3%**	**7**	**5%**	**<0.001***	23	1.6%	6	1.7%	**0.036***	26	1.7%	3	1.4%	**0.001***
	**Few times/week**	263	16.1%	18	12.8%	**Cramer's V=0.158**	224	15.7%	63	18.1%	**Cramer's V=0.085**	239	15.6%	38	17.1%	**Cramer's V=0.121**
	**Once/day**	**37**	**2.3%**	**7**	**5%**		29	2%	13	3.7%		**32**	**2.1%**	**11**	**5%**	
	**Few times/day**	**4**	**0.2%**	**5**	**3.5%**		**4**	**0.3%**	**5**	**1.4%**		**3**	**0.2%**	**5**	**2.3%**	

*Fisher's Exact Test, statistical significance (*p*<0.05).
