# Supplementary material for: Parent-Reported Oral Health Status and Dental Care Habits in Primary Schoolchildren: A Cross-Sectional Study
Source: Children (Basel). 2026 Oct 2;13(10):1342. doi: 10.3390/children13101342 (PMC13703419; doi:10.3390/children13101342)
Supplement: Supplementary file 1 [file children-13-01342-s001.zip › children-4529769-supplementary/Table S3- Supplementary Material.pdf]

Table S3. GEE models used in the manuscript.

Model 1 – Dependent variable – Tough food difficulties, Independent variable – Age

Parameter*	OR (95% C.I.)
**Age**	0.908 (0.878-0.939), p<0.001

*Model – Generalized estimating equation, Structure – Binomial, Link function - Logit, Correlation Matrix Structure – Independent, Covariance estimator – Robust, Clustering variable – Children class, Number of clusters – 5, Dependent variable coding – Absent (0) (Reference) and Present (1). N=1773

Model 2 – Dependent variable – Soft drink consumption, Independent variable – Age

Parameter*	OR (95% C.I.)
**Age**	1.113 (1.053-1.177), p<0.001

*Model – Generalized estimating equation, Structure – Multinomial, Link function - Cumulative logit (Ordinal), Correlation Matrix Structure – Independent, Covariance estimator – Robust, Clustering variable – Children class, Number of clusters – 5, Dependent variable coding (Ascending order) – Never (1), Few times/month (2), Once/week (3), Few times/week (4), Once/day (5), Few times/day (6). N=1870

Model 3 – Dependent variable – Dental health status, Independent variables – Child age, Gender, Environment, Parent education, Dental hygiene, Fluoride toothpaste usage, Biscuits, cakes, cream cakes, sweet pies, etc., consumption, Soft drinks consumption

Parameter*	OR (95% C.I.)
**Age**	1.026 (0.932-1.130), p=0.598
**Gender (Son) (Daughter – Reference)**	0.836 (0.668-1.047), p=0.118
**Environment (Urban) (Rural – Reference)**	1.054 (0.882-1.259), p=0.566
**Father's education (Academic) (Non-academic – Reference)**	1.417 (1.082-1.855), p=0.011
**Mother's education (Academic) (Non-academic – Reference)**	1.017 (0.854-1.212), p=0.851
Dental hygiene frequency	
**Never (Reference)**	-
**Few times/month**	30.754 (1.559-606.545), p=0.024
**Once/week**	21.107 (2.050-217.331), p=0.010
**Few times/week**	16.808 (1.432-197.220), p=0.025
**Once/day**	28.398 (2.539-317.643), p=0.007

**Twice or more/day**	67.119 (4.878-923.501), p=0.002
**Fluoride toothpaste usage (Present) (Absent – Reference)**	0.967 (0.768-1.218), p=0.775
Biscuits, cakes, cream cakes, sweet pies, etc., consumption	
**Never (Reference)**	-
**Few times/month**	1.249 (0.928-1.681), p=0.142
**Once/week**	1.569 (1.268-1.942), p<0.001
**Few times/week**	1.250 (0.891-1.752), p=0.196
**Once/day**	0.888 (0.699-1.127), p=0.329
**Few times/day**	0.663 (0.439-1.002), p=0.051
Soft drinks consumption	
**Never (Reference)**	-
**Few times/month**	0.957 (0.740-1.237), p=0.735
**Once/week**	0.850 (0.659-1.096), p=0.211
**Few times/week**	0.898 (0.629-1.281), p=0.551
**Once/day**	0.851 (0.683-1.061), p=0.151
**Few times/day**	0.683 (0.425-1.098), p=0.115

*Model – Generalized estimating equation, Structure – Multinomial, Link function - Cumulative logit (Ordinal), Correlation Matrix Structure – Independent, Covariance estimator – Robust, Clustering variable – Children class, Number of clusters – 5, Dependent variable coding (Ascending order) – Poor (1), Average (2), Good (3), Very good (4), Excellent (5). N=1358

Model 4 – Dependent variable – Gum health status, Independent variables – Child age, Gender, Environment, Parent education, Dental hygiene, Fluoride toothpaste usage, Biscuits, cakes, cream cakes, sweet pies, etc., consumption, Soft drinks consumption

Parameter*	OR (95% C.I.)
**Age**	0.965 (0.884-1.054), p=0.430
**Gender (Son) (Daughter – Reference)**	0.788 (0.585-1.062), p=0.118
**Environment (Urban) (Rural – Reference)**	1.062 (0.955-1.181), p=0.269
**Father's education (Academic) (Non-academic – Reference)**	1.786 (1.228-2.598), p=0.002
**Mother's education (Academic) (Non-academic – Reference)**	0.945 (0.707-1.263), p=0.701
Dental hygiene frequency	
**Never (Reference)**	-

**Few times/month**	32.103 (4.054-254.221), p=0.001
**Once/week**	13.748 (1.848-102.301), p=0.010
**Few times/week**	14.017 (2.955-66.494), p=0.001
**Once/day**	20.337 (3.753-110.197), p<0.001
**Twice or more/day**	38.792 (5.759-261.304), p<0.001
**Fluoride toothpaste usage (Present) (Absent – Reference)**	1.006 (0.809-1.251), p=0.957
Biscuits, cakes, cream cakes, sweet pies, etc., consumption	
**Never (Reference)**	-
**Few times/month**	0.509 (0.183-1.417), p=0.196
**Once/week**	0.545 (0.226-1.315), p=0.177
**Few times/week**	0.546 (0.254-1.177), p=0.122
**Once/day**	0.442 (0.222-0.879), p=0.020
**Few times/day**	0.422 (0.179-0.993), p=0.048
Soft drinks consumption	
**Never (Reference)**	-
**Few times/month**	0.756 (0.556-1.029), p=0.076
**Once/week**	0.620 (0.476-0.806), p<0.001
**Few times/week**	0.636 (0.504-0.802), p<0.001
**Once/day**	0.528 (0.348-0.801), p=0.003
**Few times/day**	0.571 (0.380-0.858), p=0.007

*Model – Generalized estimating equation, Structure – Multinomial, Link function - Cumulative logit (Ordinal), Correlation Matrix Structure – Independent, Covariance estimator – Robust, Clustering variable – Children class, Number of clusters – 5, Dependent variable coding (Ascending order) – Poor (1), Average (2), Good (3), Very good (4), Excellent (5). N=1370

Model 5 – Dependent variable – Dental pain frequency Independent variables – Child age, Gender, Environment, Parent education, Dental hygiene, Fluoride toothpaste usage, Biscuits, cakes, cream cakes, sweet pies, etc., consumption, Soft drinks consumption

**Parameter***	**OR (95% C.I.)**
**Age**	0.991 (0.909-1.081), p=0.836
**Gender (Son) (Daughter – Reference)**	0.830 (0.700-0.985), p=0.033
**Environment (Urban) (Rural – Reference)**	0.962 (0.782-1.183), p=0.713
**Father's education (Academic) (Non-academic – Reference)**	0.854 (0.649-1.123), p=0.258
**Mother's education (Academic) (Non-academic – Reference)**	1.242 (0.971-1.588), p=0.084
Dental hygiene frequency	
**Never (Reference)**	-
**Few times/month**	0.520 (0.170-1.518), p=0.232
**Once/week**	0.529 (0.060-4.695), p=0.568
**Few times/week**	0.409 (0.146-1.146), p=0.089
**Once/day**	0.313 (0.098-0.999), p=0.049
**Twice or more/day**	0.182 (0.054-0.617), p=0.006
**Fluoride toothpaste usage (Present) (Absent – Reference)**	0.958 (0.885-1.038), p=0.296
Biscuits, cakes, cream cakes, sweet pies, etc., consumption	
**Never (Reference)**	-
**Few times/month**	0.128 (0.016-1.041), p=0.055
**Once/week**	0.098 (0.011-0.884), p=0.038
**Few times/week**	0.107 (0.013-0.913), p=0.041
**Once/day**	0.134 (0.016-1.121), p=0.064
**Few times/day**	0.193 (0.025-1.467), p=0.112
Soft drinks consumption	
**Never (Reference)**	-
**Few times/month**	1.319 (0.934-1.864), p=0.116
**Once/week**	1.911 (1.261-2.896), p=0.002
**Few times/week**	1.910 (1.452-2.512), p<0.001
**Once/day**	1.575 (1.076-2.306), p=0.019
**Few times/day**	2.647 (1.597-4.386), p<0.001

*Model – Generalized estimating equation, Structure – Multinomial, Link function - Cumulative logit (Ordinal), Correlation Matrix Structure – Independent, Covariance estimator – Robust, Clustering variable – Children class, Number of clusters – 5, Dependent variable coding (Ascending order) – Never (1), Rarely (2), Occasionally (3), Often (4). N=1393

Model 6 – Dependent variable – Dental visit frequency, Independent variables – Child age, Gender, Environment, Parent education, Fluoride toothpaste usage, Biscuits, cakes, cream cakes, sweet pies, etc., consumption, Soft drinks consumption (Dental hygiene was omitted due to quasi-complete separation that occurred in the model)

Parameter*	OR (95% C.I.)
**Age**	1.019 (0.941-1.103), p=0.647
**Gender (Son) (Daughter – Reference)**	0.935 (0.809-1.082), p=0.367
**Environment (Urban) (Rural – Reference)**	1.095 (0.793-1.511), p=0.582
**Father's education (Academic) (Non-academic – Reference)**	1.168 (1.002-1.361), p=0.048
**Mother's education (Academic) (Non-academic – Reference)**	2.223 (1.914-2.583), p<0.001
**Fluoride toothpaste usage (Present) (Absent – Reference)**	1.324 (1.141-1.536), p<0.001
Biscuits, cakes, cream cakes, sweet pies, etc., consumption	
**Never (Reference)**	-
**Few times/month**	0.215 (0.017-2.675), p=0.232
**Once/week**	0.232 (0.021-2.531), p=0.231
**Few times/week**	0.223 (0.022-2.227), p=0.201
**Once/day**	0.262 (0.024-2.917), p=0.276
**Few times/day**	0.291 (0.028-3.032), p=0.302
Soft drinks consumption	
**Never (Reference)**	-
**Few times/month**	1.146 (0.754-1.743), p=0.523
**Once/week**	1.101 (0.703-1.724), p=0.675
**Few times/week**	1.071 (0.764-1.502), p=0.691
**Once/day**	0.882 (0.495-1.572), p=0.669
**Few times/day**	0.747 (0.457-1.223), p=0.247

*Model – Generalized estimating equation, Structure – Multinomial, Link function - Cumulative logit (Ordinal), Correlation Matrix Structure – Independent, Covariance estimator – Robust, Clustering variable – Children class, Number of clusters – 5, Dependent

variable coding (Ascending order) – Never visited a dentist (0), Never visited a dentist in the last 12 months (1), Once (2), Twice (3), Three times (4), Four times (5), Five times or more (6). N=1402

Model 7 – Dependent variable – Reason for last dental visit, Independent variables – Child age, Gender, Environment, Parent education, Dental hygiene, Fluoride toothpaste usage, Soft drinks consumption (Biscuits, cakes, cream cakes, sweet pies, etc., were omitted due to quasi-complete separation that occurred in the model)

Dental visit reason	Variable	OR (95% C.I.)
Routine check-up	**Age**	1.092 (0.994-1.200), p=0.066
Treatment		1.053 (0.953-1.164), p=0.307
Dental pain		0.870 (0.791-0.956), p=0.004
Routine check-up	**Gender (Son) (Reference = Daughter)**	1.080 (0.828-1.410), p=0.570
Treatment		0.754 (0.560-1.014), p=0.061
Dental pain		1.193 (0.907-1.571), p=0.206
Routine check-up	**Environment (Urban) (Reference = Rural)**	1.252 (0.942-1.663), p=0.121
Treatment		0.954 (0.697-1.306), p=0.770
Dental pain		0.826 (0.617-1.105), p=0.198
Routine check-up	**Father's education (Academic) (Reference = Non-academic)**	0.982 (0.707-1.363), p=0.912
Treatment		1.009 (0.703-1.446), p=0.962
Dental pain		1.000 (0.705-1.417), p=0.999
Routine check-up	**Mother's education (Academic) (Reference = Non-academic)**	1.061 (0.764-1.472), p=0.724
Treatment		1.443 (1.001-2.082), p=0.049
Dental pain		0.682 (0.487-0.957), p=0.026
Routine check-up	**Dental hygiene frequency (Once/week) (Reference = Few times/month)**	0.545 (0.071-4.169), p=0.558
Treatment		3.902 (0.352-43.205), p=0.267
Dental pain		0.714 (0.127-4.024), p=0.702
Routine check-up	**Dental hygiene frequency (Few times/week) (Reference = Few times/month)**	0.807 (0.194-3.355), p=0.767
Treatment		1.986 (0.250-15.772), p=0.516
Dental pain		0.892 (0.237-3.358), p=0.865
Routine check-up	**Dental hygiene frequency (Once/day) (Reference = Few times/month)**	1.262 (0.333-4.780), p=0.731
Treatment		2.856 (0.394-20.705), p=0.299

Dental pain		0.489 (0.141-1.697), p=0.259
Routine check-up	**Dental hygiene frequency (Two or more/day) (Reference = Few times/month)**	2.133 (0.564-8.072), p=0.264
Treatment		3.075 (0.423-22.358), p=0.267
Dental pain		0.260 (0.075-0.904), p=0.034
Routine check-up	**Fluoride toothpaste usage (Present) (Reference = Absent)**	1.020 (0.766-1.358), p=0.893
Treatment		0.982 (0.714-1.350), p=0.910
Dental pain		0.999 (0.743-1.343), p=0.995
Routine check-up	**Soft drinks consumption (Few times/month) (Reference = Never)**	0.365 (0.229-0.584), p<0.001
Treatment		1.629 (0.948-2.799), p=0.077
Dental pain		2.257 (1.278-3.985), p=0.005
Routine check-up	**Soft drinks consumption (Once/week) (Reference = Never)**	0.399 (0.232-0.685), p<0.001
Treatment		1.489 (0.803-2.763), p=0.206
Dental pain		2.239 (1.183-4.238), p=0.013
Routine check-up	**Soft drinks consumption (Few times/week) (Reference = Never)**	0.438 (0.268-0.716), p=0.001
Treatment		1.410 (0.799-2.491), p=0.235
Dental pain		2.120 (1.179-3.813), p=0.012
Routine check-up	**Soft drinks consumption (Once/day) (Reference = Never)**	0.537 (0.297-0.968), p=0.038
Treatment		0.660 (0.300-1.455), p=0.303
Dental pain		2.797 (1.429-5.476), p=0.002
Routine check-up	**Soft drinks consumption (Few times/day) (Reference = Never)**	0.322 (0.161-0.645), p=0.001
Treatment		0.841 (0.364-1.942), p=0.684
Dental pain		3.939 (1.900-8.163), p<0.001

*Model – Generalized estimating equation, Structure – Multinomial, Link function - Logit, Correlation Matrix Structure – Independent, Covariance estimator – Robust, Clustering variable – Children class, Number of clusters – 5, Dependent variable coding – Routine check-up (1), Treatment (2), Dental pain (3). Reference category is established for each category as the non-selection of the specific category. N=988
